# Therapeutic Implications of Targeting Energy Metabolism in Breast Cancer

**DOI:** 10.1155/2013/109285

**Published:** 2013-02-03

**Authors:** Meena K. Sakharkar, Babita Shashni, Karun Sharma, Sarinder K. Dhillon, Prabhakar R. Ranjekar, Kishore R. Sakharkar

**Affiliations:** ^1^Graduate School of Life and Environmental Sciences, University of Tsukuba, Tennoudai 1-1-1, Tsukuba 305-8572, Japan; ^2^Institute of Biological Sciences, Faculty of Science, University of Malaya, 50603 Kuala Lumpur, Malaysia; ^3^IRSHA, Bharati Vidyapeeth University, Pune 411043, India; ^4^Omicsvista, Singapore 120417; ^5^Rajiv Gandhi Institute of Information Technology and Biotechnology, Bharati Vidyapeeth University, Pune 411046, India

## Abstract

PPARs are ligand activated transcription factors. PPAR**γ** agonists have been reported as a new and potentially efficacious treatment of inflammation, diabetes, obesity, cancer, AD, and schizophrenia. Since cancer cells show dysregulation of glycolysis they are potentially manageable through changes in metabolic environment. Interestingly, several of the genes involved in maintaining the metabolic environment and the central energy generation pathway are regulated or predicted to be regulated by PPAR**γ**. The use of synthetic PPAR**γ** ligands as drugs and their recent withdrawal/restricted usage highlight the lack of understanding of the molecular basis of these drugs, their off-target effects, and their network. These data further underscores the complexity of nuclear receptor signalling mechanisms. This paper will discuss the function and role of PPAR**γ** in energy metabolism and cancer biology in general and its emergence as a promising therapeutic target in breast cancer.

## 1. Introduction

**Figure 1:**
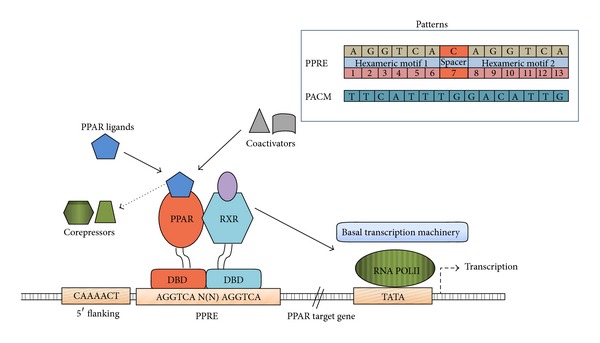
PPAR gamma activation mechanism. PPRE and PACM motifs are shown.

The peroxisome proliferator-activated receptors (PPARs) are ligand activated transcription factors, belonging to the nuclear receptor superfamily, that control the expression of genes involved in organogenesis, inflammation, cell differentiation, proliferation, lipid, and carbohydrate metabolism [1, 2]. PPARs activated by their selected ligands, heterodimerizes and its receptor with the 9-cis-retinoic acid receptor, they then bind to peroxisome proliferator response elements (PPREs), specific sequences in their target genes. The consensus PPRE site consists of a direct repeat of the sequence AGGTCA separated by a single/double nucleotide, which is designated as DR-1 site/DR-2 site [3] (Figure 1). Each major isoforms of PPAR (PPAR*α*, PPAR*β*/*δ*, and PPAR*γ*), encoded by a different gene, performs different functions and exhibit different tissue localizations in many parts of the human body [4]. The peroxisome proliferator-activated receptor *γ* (PPAR*γ*) is the most extensively studied subtype of the PPARs [5]. PPAR*γ* is expressed in adipose tissue, colon, immune system, hematopoietic cells, and retina involved in lipid anabolism, adipocyte differentiation, control of inflammation, macrophage maturation, embryo implantation, and molecular targets of antidiabetic thiazolidinediones [6]. Its role in cancer development and potential as a target for cancer prevention and treatment strategies has been noted in recent years. Activation of PPAR*γ* could possibly be an approach to induce differentiation in cells thereby inhibiting proliferation of a variety of cancers. This antiproliferative effect has been reported in many different cancer cell lines including breast[7],colon[8], prostate[9], and non-small-cell lung cancer[10]. In particular, breast tissue was found to express PPAR*γ* in amounts greater than those found in normal breast epithelium. Ligand activated PPAR*γ* is reported to inhibit invasion and metastasis of breast cancer cells and induce G1/S arrest by upregulation of p21^WAF1^/Cip1or p27^Kip1^, and downregulation of cyclin D1 [11–13]. Moreover, PPAR*γ* on activation by specific ligands exerts antitumor activity through growth inhibition and cellular differentiation [14–17]. Imbalances in expression of target genes forms the core of metabolic syndrome and cancer regulation through atherogenic metabolic triad/lipid triad metabolism modulation by PPARs [18]. Despite these promising results, the target genes involved in the anticancer activity of PPAR*γ* ligands and their pathways still remain elusive. 

**Figure 2:**
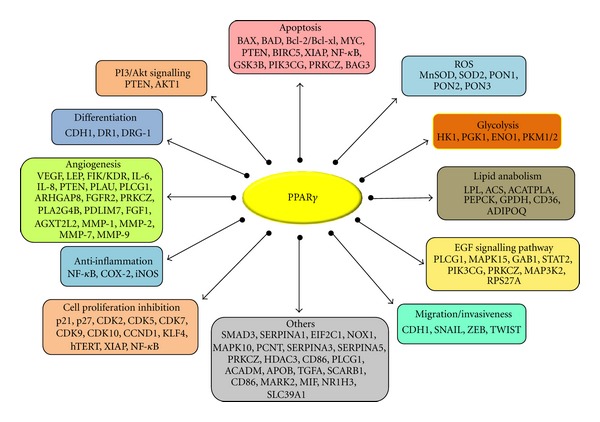
PPAR gamma gene targets and their pathways.

Breast cancer is the fifth most common cancer globally and accounts for the highest morbidity and mortality. It is the second highest occurring cancer in women and one of the leading causes of death[19]. Although antiestrogens have provided an effective endocrine therapy, a significant proportion of patients have acquired resistance to these drugs, others are intrinsically resistant [20]. Hence, there is a requirement for alternative therapeutics to treat breast cancer. Development of selective anticancer agents based on the biological differences between normal and cancer cells is essential to improve therapeutic selectivity, sensitivity, and specificity. A list of genes reported in the literature to be regulated by PPAR*γ* and involved in breast cancer is shown in Figure 2. 

Differences in energy metabolism between normal and cancer cells are reported andalterations in cellular bioenergetics are one of the hallmarks of cancer [21]. The general principles of metabolic control analysis can be effective for cancer management as abnormal energy metabolism and biological disorder are characteristics of tumors [22]. In line with this, increased aerobic glycolysis and elevated oxidative stress are two prominent biochemical features frequently observed in cancer cells, as shown by the Warburg hypothesis. This paper will discuss the function and role of PPAR*γ* in energy metabolism and cancer biology in general and its emergence as a promising therapeutic target in breast cancer. 

## 2. Glycolysis and Cancer 

**Figure 3:**
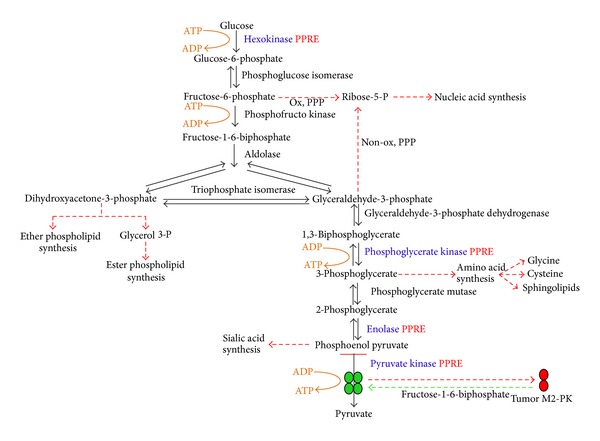
Tumor cells have altered glucose metabolism. Many glycolytic enzymes are ubiquitously expressed in cancers. One such glycolytic enzyme is pyruvate kinase type M2 whose levels are found to be elevated in human cancer biopsies, compared to adjacent normal tissues. PKM2 is a key regulator of the metabolic budget system in tumor cells which promotes the Warburg effect and tumor growth. This tumor specific PKM2 can be switched between dimeric and tetrameric forms in cancer cells. Dimeric PKM2 has a higher *K*_*m*_ value for the substrate PEP than the tetrameric form of PKM2 and is inactive at physiological concentrations of PEP. PKM2 is allosterically activated by the glycolytic metabolite fructose-1,6-biphosphate (FBP) and serine. This leads to accumulation of energy rich phospho metabolites upstream of glycolytic pathway which are then channelled to macromolecule biosynthesis via pentose phosphate pathway (PPP). These pathways include pyrimidine, glycerol, and serine/glycine biosynthesis (red arrows) instead of leading to oxidative metabolism for energy production thereby promoting cancer cell proliferation and tumor growth.

Coordinated upregulation of glycolysis pathway proteins has been detected in several different tumor types including breast cancer tumors [23–26]. Amon et al. identified increased levels of glycolysis proteins in plasmas of women with breast cancer [27]. Glycolysis for ATP synthesis rather than oxidative phosphorylation occurs primarily when cells are deprived of oxygen, but the Warburg hypothesis suggests the central role of glycolysis in cancer and tumor cells even in the presence of oxygen [28]. Warburg determined that there is a tenfold increase of glucose consumption in cancer cells as compared to normal cells, and a twofold production of lactic acid as compared to that produced by normal tissue. Cancer cells are provided with several growth advantages like growth of cells in adverse microenvironment, generation of substrates for glycosylation reactions, and supply of precursors for biosynthetic reactions by aerobic glycolysis/enhanced glucose uptake [29, 30]. Recent reports indicate that mTOR activation is a key regulator of the Warburg effect leading to upregulation of glycolytic enzymes [31, 32]. Aerobic glycolysis is disadvantageous and detrimental as compared to oxidative phosphorylation due to the low ATP yield (only 2 mol ATP/mole of glucose while oxidative metabolism of glucose results in about 36 mol ATP/mole of glucose) as compared to investment as well as lactic acidosis that may result from Cori's cycle that follows aerobic glycolysis resulting in release of proteolytic enzymes and therefore local toxicity including cell death and extracellular matrix degradation [33, 34]. A recent report suggests that a relatively minor fraction (<30%) of a cancer cell's aerobic ATP production is derived from glycolysis according to mass balance analyses [35]. Conflicting evidence suggests that hydrogen ions production by glycolysis create the acidic environment responsible for degradation of the extracellular matrix, critical for facilitating tumor invasion into normal host tissue [36]. Adaptive advantages are also conferred by increased glycolysis (or pentose phosphate metabolism) if it allows excess pyruvate to be available for lipid synthesis or providing essential anabolic substrates, such as ribose for nucleic acid synthesis [37]. Glucose consumption through the pentose pathway may also provide essential reducing equivalents (NADPH) to reduce the toxicity of reactive oxygen species conferring resistance to senescence and anabolic substrates such as ribose for nucleic acid synthesis [38]. These evolutionary advantages can explain the remarkable prevalence of the glycolytic phenotype in human cancers and the otherwise puzzling observation that malignant cells remain glycolytic even in the presence of normoxia. This conceptual model of constitutive upregulation of glycolysis has been demonstrated by empirical studies and is consistently observed during the transition from premalignant lesions and invasive cancer [39, 40]. It is interesting to note that several of the glycolytic enzymes have isoforms which are expressed only in malignant cells and thus can be potential targets for therapy (Figure 3). This is important since aerobic glycolysis is an existing metabolic function in all eukaryotic cells and using normal isoforms as target for cancer therapeutics may lead to cytotoxicity issues. Interestingly, hypoxia-mediated HIF-1 has been suggested to lead to the expression of specific isoforms of glycolytic enzymes and transporters through alternative splicing. Pre-mRNA splicing has been reported to play a core role in “orchestrating” cellular stress phrase to gene-expression profiles [41]. A multiphasic response including increased expression of components of the glycolytic pathways including membrane glucose transporters in a HIF-1-dependent manner is elicited by upregulation of the HIF system [42]. Concurrently,PPAR*γ* activates a number of genes in tissues increasing glucose and lipid uptake and glucose oxidation, simultaneously decreasing free fatty acid concentration and insulin resistance. Hence, targeted therapies may be eventually led by understanding the molecular and physiological causes and consequences of upregulated glycolysis and modulation of these. In line with the above, cancer treatment strategies through the target of energy metabolism of cancer could include glucose deprivation, inhibition of the glycolytic pathway (3-bromopyruvate (3-BrPA)), glucose analogues (2-deoxyglucose (2DG)), inhibition of glucose transport (Imatinib), and exploitation of HIF (PX-478). 

Here, it must be mentioned that glycolytic inhibitors in combination with other therapies have proven to be more promising than being alone as tumor cells and tumor microenvironment are very heterogeneous and cells within an invasive cancer may use a range of metabolic pathways including some in which oxidative metabolism of glucose or fatty acids contributes significantly to ATP production [43]. Moreover, enhanced glycolysis may be possible via different mechanisms such as gene amplification, increased gene expression, increased translation, posttranslational modification, and regulation by protein-protein interactions in the cytoplasm. Different cancers exploit different mechanisms to achieve increased glucose consumption. 

## 3. Isoforms of Glycolytic Genes and PPAR*γ*

Cancer cells are found to upregulate glucose transport and switch their main energy supply pathway from oxidative phosphorylation to glycolysis depending heavily on glucose as both energy and biosynthesis sources. Thus, cancer cells are more sensitive than normal cells to changes in glucose concentration [44, 45] and it is easier than normal cells to induce death in limited glucose supply and disruption of glycolysis [46]. These molecular and metabolic changes also provide targets for cancer treatment. Inhibiting either various steps of glycolysis or glucose transport, the first rate-limiting step leading to glycolysis, is likely to severely disrupt both energy supply and biosynthesis processes inside cancer cells, resulting in reduction of proliferation rate and induction of apoptosis of cancer cells. Several glycolytic enzymes are predicted by us to have the PPRE site, suggesting their regulation by PPAR*γ* (Figure 3).

The enzymes in glycolytic pathway and their isoforms are discussed below.

### 3.1. Hexokinases

 They catalyze the first irreversible step of the glycolytic pathway for the phosphorylation of glucose to glucose-6-phosphate with consumption of ATP. Four important mammalian hexokinase isozymes that vary in subcellular locations and kinetics with respect to different substrates and conditions and physiological functions are known and designated as HK1-4 [47]. HK2, the predominant isoform overexpressed in malignant tumors, strategically binds to the outer mitochondrial membrane coupling ATP formation in mitochondria to the phosphorylation of glucose, thus conferring cancer cells with a highly glycolytic phenotype and ample biosynthetic precursor [48, 49]. In addition to its critical metabolic role, HK2 can also promote cancer by repressing mitochondrial function on cell death, immortalizing cancer cells.

### 3.2. Fructose-2, 6-Bisphosphatase

 6-phosphofructo-2-kinase/fructose-2, 6-bisphosphatase 3 (PFKFB3), is a bifunctional enzyme and is central to glycolytic flux. It is downstream to the metabolic stress sensor AMP-activated protein kinase (AMPK) that modulates glycolysis and possibly activates isoforms of PFKFB, specifically PFKFB3 expressed in tumor cells. It has been demonstrated that long-term low pH exposure induces AMPK activation, which results in the upregulation of PFKFB3 and an increase in its serine residue phosphorylation. Pharmacologic activation of AMPK is responsible for increase in PFKFB3 as well as an increase in glucose consumption, whereas inhibition of AMPK results in the downregulation of PFKFB3 and decreased glycolysis [50].

### 3.3. Pyruvate Kinase (PKM)

It converts phosphoenolpyruvate to pyruvate and regulates the rate-limiting final step of glycolysis. It has two specific isoforms: the adult isoform, PKM1, promotes oxidative phosphorylation and the PKM2 isoform, which promotes aerobic glycolysis and is expressed in embryonic and cancerous cells. The above isoforms are produced as a result of mutually exclusive alternative splicing of the PKM pre-mRNA that corresponds to inclusion of either exon 9 (PKM1) or exon 10 (PKM2) [51, 52]. We have recently shown that PKM2 is downregulated by PPAR*γ* activation by 15d-PGJ_2_, a PPAR-gamma ligand [53]. Concurrently, PGK1, another important enzyme at ATP generation step in glycolysis, has been reported by us to be downregulated by PPAR gamma upon activation by 15d-PGJ_2_ [54].

## 4. pH Regulator NHE1

Cancer cells thrive in anacidic environment and do not survive in normal or more alkaline environment. Lactic acid production due to increased glycolysis by cancer cells makes the environment even more acidic. NHE1 is a ubiquitously expressed membrane phosphoglycoprotein comprising of 10–12 transmembrane segments (N-terminal) and a large cytoplasmic tail. NHE1 is reported to be involved in intracellular pH (pHi) homeostasis and cell volume regulation [55, 56]. Under physiological conditions, the Na^+^/H^+^ exchanger NHE1 extrudes one H^+^ ion in exchange for one extracellular Na^+^ ion. An alkaline pHi together with an acidic extracellular environment is associated with a transformed or tumorigenic phenotype, suggesting that active proton extrusion capabilities provide a twofold advantage to tumor cells: the alkaline pHi favours metabolic processes associated with cellular proliferation, whereas the acidic extracellular environment, a consequence of H^+^ extrusion, enhances the invasive capacity of transformed cells [57]. The role of pHi and NHE1 in the regulation of tumorigenic and metastatic properties of tumor cells, however, remains unclear. Since NHE1 is activated upon growth factor stimulation, it has been suggested that NHE1 also plays a role in cellular proliferation [58]. In addition, tumor cells deficient in NHE1 activity either do not grow or show severely arrested growth when implanted in immunodeficient mice [59, 60].

Interestingly, a recent report implicated the pH regulator, NHE1, in tumor cell growth is arrested by activated PPAR*γ*, an interesting connotation considering that the activation of NHE1 is an oncogenic signal necessary for the development and maintenance of the transformed phenotype [57, 60, 61]. Also, it was recently reported that decrease in NHE1 expression led to tumor cell growth arrest, intracellular acidification, and sensitization to death stimuli [62]. These data support downregulation of NHE1 as a possibility for inducing growth arrest in cancer cells [9]. In light of the increased expression of PPAR*γ* in breast cancer cell lines and its association with acidic intracellular pH, we hypothesized that, in addition to inhibiting NHE1 activity, ligand-induced activation of PPAR*γ* could regulate NHE1 gene expression. Interestingly, our results corroborate with the data which reported that exposure of breast cancer cell lines expressing high levels of PPAR*γ* to natural ligands of PPAR*γ* significantly inhibited NHE1 gene expression compared with noncancerous cells or cancer cell lines expressing low levels of PPAR*γ* [9, 63].

## 5. MnSOD as a Target for PPAR*γ*

Cancer cells have higher levels of reactive oxygen species (ROS) than normal cells. They exhibit increased intrinsic ROS stress, due to oncogenic stimulation, increased metabolic activity, and mitochondrial malfunction [64].The increased amounts of ROS in cancer cells may have significant consequences, such as stimulation of cellular proliferation, promotion of mutations and genetic instability, and alterations in cellular sensitivity to anticancer agents. It is logical to speculate that the biochemical and molecular changes caused by ROS may contribute to the development of a heterogeneous cancer cell population and the emergence of drug-resistant cells during disease progression.Cells have evolved several antioxidant defenses, including repair and detoxifying enzymes, and small scavenger molecules, such as glutathione. The intracellular ROS-scavenging system includes superoxide dismutases (SOD), glutathione peroxidase (GPx), peroxiredoxins (PRDXs), glutaredoxins, thioredoxins (TRXs), and catalases. In mitochondria, superoxide anion (O_2_^−^) can be dismutated to hydrogen peroxide (H_2_O_2_) by two enzymes, namely, copper-zinc superoxide dismutase (CuZnSOD) and manganese superoxide dismutase (MnSOD), that are present in the mitochondrial matrix and in the intermembrane space, respectively [65]. Once generated, H_2_O_2_ can be quenched by GPx in mitochondria, or by catalase in the cytosol. The expression of antioxidant enzymes is regulated by complex mechanisms, oxidative stress being a major factor that induces the adaptive expression of these enzymes [66]. Thus, increased ROS stress in cancer cells is likely to cause increased expression of SOD and other antioxidant enzymes. In fact, analysis of SOD protein expression in primary tissues from adenocarcinomas of the stomach and squamous cell carcinomas of the oesophagus showed a significantly higher MnSOD expression in the cancer cells compared to normal mucosa cells [67]. The activities of SOD, glutathione peroxidase (GPx), and glutathione-*S*-transferase (GST) were increased significantly in the mitochondria of colorectal cancer tissues compared to adjacent normal tissues of the same subjects [68]. Increased SOD levels were also observed in breast cancer tissue from 23 patients [69]. Detection of MnSOD using specific antibody showed positive reactions with ovarian carcinomas and malignant brain tumors, but not with the respective normal control tissues [70]. Some studies further demonstrated a significantly increased expression of CuZnSOD (SOD1), MnSOD (SOD2), and catalase in chronic lymphocytic leukemia cells and ovarian cancer cells [71]. Increases in SOD1 and SOD2 have been observed in blood samples from patients with various types of leukemia [72]. Interestingly, leukemia regression was accompanied by a decrease in the serum MnSOD, suggesting that MnSOD in serum may serve as an indicator of disease activity. Analysis of SOD in blood samples from patients with ovarian cancer yielded conflicting results [73]. We reported human MnSOD as a PPAR*γ* target gene and the downregulation of MnSOD gene expression *in vitro* by PPAR*γ* agonists [9]. 

## 6. Glycolysis, Diabetes, Obesity, Cancer, and PPAR*γ*

The nuclear transcription factor PPAR*γ* is highly expressed in adipose tissue and plays a central role in adipocyte function, fat storage, and lipid metabolism. PPAR*γ* activators are commonly used to treat patients with type 2 diabetes who share metabolic abnormalities including excessive and inflamed adipose tissue [74], particularly in visceral depots [75], elevated circulating concentrations of nonesterified fatty acids (NEFA), triglycerides, glucose, insulin, and inflammatory mediators and reduced concentrations of adiponectin and high-density lipoprotein cholesterol (HDL-C). On the other hand, key cancer-related oncogenes type I receptor tyrosine kinase HER2- (*erb*B-2-) dependent endogenous fatty acid (FA) constitutive upregulation and biogenesis catalyzed by lipogenic enzymes such as fatty acid synthase (FASN) [76, 77], constitutes an oncogenic stimulus that drives normal epithelial cells progression toward malignancyand has been reported to provide a “lipogenic benefit” in terms of enhanced breast cancer cell proliferation, survival, chemoresistance, and metastasis [78]. This would require a constant supply of precursors, energy, and reducing equivalent required for FASN-driven lipogenesis. Moreover, excess of the end product of *de novo* FA synthesis, namely, palmitate (and other palmitate-like saturated FAs), is toxic to cells because of its ability to generate a variety of apoptotic signals involving either the production of ROS or the synthesis of ceramide. Indeed, disturbance in lipogenic balance caused by the accumulation of FAs and neutral lipids in nonadipose tissue stimulates lipolysis and apoptosis[79, 80]. Moreover, palmitate excess could have feedback to inhibit endogenous FA synthesis [81]. Also, PPAR*γ*, in HER2-positive cancer cells, which produce high levels of endogenous fat, helps to convert FAs to triglycerides, thus allowing these cells to avert the cell death resulting from endogenous palmitate-related lipotoxicity [82]. It must be noted here that PPAR*γ* has been reported to directly interact with SIRT1 (human silent information regulator type 1-deacetylase protein) and inhibit SIRT1 activity, forming a negative feedback and self-regulation loop. Also, the transcriptional activity of PPAR*γ* is regulated through deacetylation by SIRT1. This association of SIRT1 and PPAR*γ* together with the transcriptional modulation of SIRT1 appears to be important in the senescence/aging process controlled by PPAR*γ* [83].

### 6.1. Diabetes, Obesity, Cancer, and PPAR*γ*

Epidemiologic evidence suggests that people with diabetes are at significantly higher risk for many forms of cancer [84]. Earlier on, Liao et al. has indicated that diabetes can be considered as a risk factor for breast cancer [85]. Boyle et al. recently investigated the association between occurrence of diabetes and breast cancer risk and observed that the risk of breast cancer in women with type 2 diabetes is increased by 27%, a figure that decreased to 16%after adjustment [86]. 

**Figure 4:**
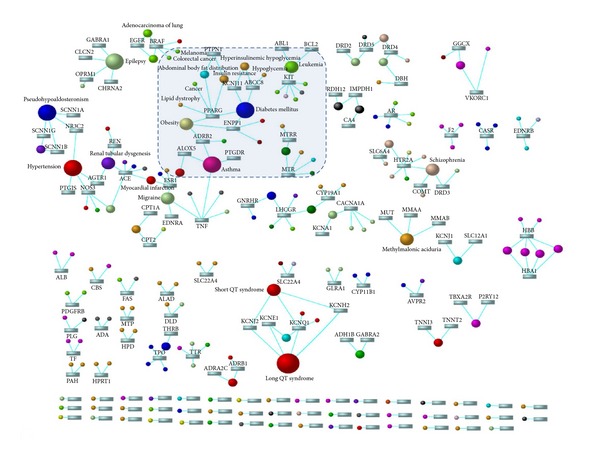
PPAR gamma disease gene network. Rectangles represent genes and circles represent diseases. Only genes involved in more than one disease are shown.

Also, obesity has been reported as a risk factor for breast cancer [87]. Interestingly, obesity is also a risk factor for type-2 diabetes [87] may be due to link between diabetes and cancer in metabolic syndrome. However, the physiological mechanism by which these risk factors interact to promote tumorigenesis is not understood. Computational analyses by us and generation of gene-disease network suggests that PPAR*γ* is implicated in pathology of several diseases including cancer, diabetes, and obesity (Figure 4). These results are in accordance with several isolated reports on their involvement in individual disorders. 

PPARs are ligand activated transcription factors and the PPAR*γ* receptor can be activated by endogenous ligands, for example, prostaglandin D2 (PGD2), 15-deoxy prostaglandin J2 (15dPGJ2), or 15-hydroxyeicosatetraenoic acid (15-HETE) [88, 89]. Synthetic ligands for PPAR*γ* include insulin sensitizing antidiabetic thiazolidinediones (TZD); troglitazone (TGZ), rosiglitazone (RGZ), ciglitazone (CGZ), or pioglitazone (PGZ) [90–92], and nonsteroidal anti-inflammatory compounds indomethacin, ibuprofen, flufenamic acid, or fenoprofen [24] are commonly known as PPAR*γ* ligands. Although, there is a rationale for the use of TZDs in patients with type 2 diabetes mellitus and they have been seen to be very effective, clinical studies have produced conflicting data, and several important aspects of PPAR*γ* action remain confusing and unresolved. Interestingly, there is no general deficiency in PPAR*γ* function in obesity or insulin-resistant states. Hence, it is not clear why synthetic activation of a receptor should give such dramatic antidiabetic effects. Moreover, variations in ligand binding efficiencies of PPAR-gamma ligands do not correlate with potency of the drug (ligand) to be antidiabetic. For example, some ligands with full agonist action, like rosiglitazone, have powerful insulin sensitizing actions, while other compounds with poor agonist activities, such as the benzylindole MRL24, retain very good antidiabetic effects [93]. 

In addition to acting as insulin sensitizers, PPAR agonists mediate *in vitro* and *in vivo* pleiotropic anticancer effects.The insulin-like growth factor (IGF) system has a role in cancer development and progression and in resistance to drug-induced apoptosis.It is now well established that the IGF system is dysregulated/overactivated (resulting from receptor and ligand abnormal expression) in a variety of human malignancies. It has also been shown that a high level of circulating insulin (hyperinsulinemia) is associated with an increased risk for a number of malignancies [94]. Moreover, hyperinsulinemia is closely associated with obesity and type 2 diabetes [95]. Thiazolidinediones (TZDs) downregulate both the PI3 K and the Ras pathway, which are the two main signalling pathways downstream receptors of the IGF system, and they also ameliorate insulin resistance and lower circulating levels of insulin and free IGF-I [96]. Moreover, TZDs, and other PPAR-agonists, such as the prostanoid 15d-PGJ2 [97], induce a variety of favorable changes (growth arrest, apoptosis, and/or partial redifferentiation) in several malignancies, including liposarcoma, and cancers of the breast, colon, pancreas, and prostate [98–101]. Oxidants and inflammatory mediators such as tumour necrosis factor-alpha (TNF-alpha) activate nuclear factor kappa B (NF-kappaB) and activator protein-1 (AP-1) transcription factors, and enhance the expression of both proinflammatory and protective antioxidant genes in several diseases including cancer and atherosclerosis [102, 103].NF-kappaB is reported to promote breast cancer cell migration and metastasis by inducing the expression of the chemokine receptor CXCR4 [104]. Also, NF-kappaB regulates the expression of a large number of genes, including growth factors, proinflammatory cytokines (e.g., TNFa, IL-6, and IL-1b), adhesion molecules (e.g., VCAM-1, P-selectin), and others such as iNOS and COX-2 [105]. PPAR activation by agonists is reported to regulate inflammatory responses [106], cell proliferation and differentiation, and apoptosis [107]. PPAR*γ* regulates expression (transcriptional level) of proinflammatory mediators such as inhibitor nuclear factor-*κ*B (NF-kappaB), signal transducers and activators of transcription (STAT)-1, and activating protein-1 (AP-1) activating signals [108]. Since, both PPAR*γ* activator and NF-KappaB are transcription factors, it was proposed that PPAR*γ* likely acts through physical interaction with NF-kappaB, resulting in the inhibition of transcriptional activation [109]. The suppressive action of PPAR*γ* on NF-kappaB was suggested to be related to its competition for limited availability of transcriptional coactivators [110] and it was suggested that due to this limitation of cofactors, neither NF-kappaB nor AP-1 could activate their target genes (e.g., iNOS or TNFa) [111]. Thus, transrepression appears to be one viable mechanism by which PPAR*γ* activators exert their anti-inflammatory effects on age-induced inflammation and oxidative stress via the downregulation of NF-kappaB.

Thus, PPAR gamma agonists have been reported as new and potentially efficacious treatment of inflammation, diabetes, obesity, cancer, AD, and schizophrenia [112]. The use of synthetic PPAR*γ* ligands as drugs and their recent withdrawal/restricted usage highlight the lack of understanding of the molecular basis of these drugs, their off-target effects, and their network. These data further underscores the complexity of nuclear receptor signalling mechanisms. Thus, there is a need to continue enhancing our understanding of the complexities of nuclear receptor pharmacology and a need to view the functions of this family of transcription factors in detail supported by clinical trials and adverse side effects data as tenable well beyond traditional discreet categories of agonism and antagonism which will open new doors to using PPAR gamma as a target. 

## References

[1] Kota BP, Huang TH, Roufogalis BD (2005). An overview on biological mechanisms of PPARs. *Pharmacological Research*.

[2] Tous M, Ferré N, Rull A (2006). Dietary cholesterol and differential monocyte chemoattractant protein-1 gene expression in aorta and liver of apo E-deficient mice. *Biochemical and Biophysical Research Communications*.

[3] Castelein H, Gulick T, Declercq PE, Mannaerts GP, Moore DD, Baes MI (1994). The peroxisome proliferator activated receptor regulates malic enzyme gene expression. *The Journal of Biological Chemistry*.

[4] van Bilsen M, van der Vusse GJ, Gilde AJ, Lindhout M, van der Lee KAJM (2002). Peroxisome proliferator-activated receptors: lipid binding proteins controling gene expression. *Molecular and Cellular Biochemistry*.

[5] Mansure JJ, Nassim R, Kassouf W (2011). Peroxisome proliferator-activated receptor gamma in bladder cancer: a promising therapeutic target in cancer. *Cellular and Genetic Practices for Translational Medicine*.

[6] Park JI (2005). The role of 15d-PGJ2, a natural ligand for peroxisome proliferator-activated receptor *γ* (PPAR*γ*), in cancer. *Pharmacological Research*.

[7] Woo CC, Loo SY, Gee V (2011). Anticancer activity of thymoquinone in breast cancer cells: possible involvement of PPAR-*γ* pathway. *Biochemical Pharmacology*.

[8] Lu YL, Li GL, Huang HL, Zhong J, Dai LC (2010). Peroxisome proliferator-activated receptor-*γ* 34C > G polymorphism and colorectal cancer risk: a meta-analysis. *World Journal of Gastroenterology*.

[9] Venkatachalam G, Kumar AP, Yue LS, Pervaiz S, Clement MV, Sakharkar MK (2009). Computational identification and experimental validation of PPRE motifs in NHE1 and MnSOD genes of human. *BMC Genomics*.

[10] Jeong Y, Xie Y, Lee W (2012). Research resource: diagnostic and therapeutic potential of nuclear receptor expression in lung cancer. *Molecular Endocrinology*.

[11] Motomura W, Okumura T, Takahashi N, Obara T, Kohgo Y (2000). Activation of peroxisome proliferator-activated receptor *γ* by troglitazone inhibits cell growth through the increase of p27^Kip1^ in human pancreatic carcinoma cells. *Cancer Research*.

[12] Govindarajan R, Ratnasinghe L, Simmons DL (2007). Thiazolidinediones and the risk of lung, prostate, and colon cancer in patients with diabetes. *Journal of Clinical Oncology*.

[13] Yu HN, Lee YR, Noh EM (2008). Induction of G1 phase arrest and apoptosis in MDA-MB-231 breast cancer cells by troglitazone, a synthetic peroxisome proliferator-activated receptor *γ* (PPAR*γ*) ligand. *Cell Biology International*.

[14] Bonofiglio D, Aquila S, Catalano S (2006). Peroxisome proliferator-activated receptor-*γ* activates p53 gene promoter binding to the nuclear factor-*κ*B sequence in human MCF7 breast cancer cells. *Molecular Endocrinology*.

[15] Pignatelli M, Cocca C, Santos A, Perez-Castillo A (2003). Enhancement of BRCA1 gene expression by the peroxisome proliferator-activated receptor *γ* in the MCF-7 breast cancer cell line. *Oncogene*.

[16] Rubin GL, Zhao Y, Kalus AM, Simpson ER (2000). Peroxisome proliferator-activated receptor *γ* ligands inhibit estrogen biosynthesis in human breast adipose tissue: possible implications for breast cancer therapy. *Cancer Research*.

[17] Turturro F, Friday E, Fowler R, Surie D, Welbourne T (2004). Troglitazone acts on cellular pH and DNA synthesis through a peroxisome proliferator-activated receptor *γ*-independent mechanism in breast cancer-derived cell lines. *Clinical Cancer Research*.

[18] Smith AG, Muscat GE (2006). Orphan nuclear receptors: therapeutic opportunities in skeletal muscle. *American Journal of Physiology*.

[19] Mukhopadhyay S, Das SK, Mukherjee S (2004). Expression of Mn-superoxide dismutase gene in nontumorigenic and tumorigenic human mammary epithelial cells. *Journal of Biomedicine and Biotechnology*.

[20] Fedele P, Calvani N, Marino A (2012). Targeted agents to reverse resistance to endocrine therapy in metastatic breast cancer: where are we now and where are we going?. *Critical Reviews in Oncology/Hematology*.

[21] Seyfried TN, Shelton LM (2010). Cancer as a metabolic disease. *Nutrition & Metabolism*.

[22] Klement RJ, Kämmerer U (2011). Is there a role for carbohydrate restriction in the treatment and prevention of cancer?. *Nutrition & Metabolism*.

[23] Bi X, Lin Q, Foo TW, Joshi S, You T, Shen HM (2006). Proteomic analysis of colorectal cancer reveals alterations in metabolic pathways: mechanism of tumorigenesis. *Molecular & Cellular Proteomics*.

[24] Unwin RD, Craven RA, Harnden P (2003). Proteomic changes in renal cancer and co-ordinate demonstration of both the glycolytic and mitochondrial aspects of the Warburg effect. *Proteomics*.

[25] Perroud B, Lee J, Valkova N (2006). Pathway analysis of kidney cancer using proteomics and metabolic profiling. *Molecular Cancer*.

[26] Isidoro A, Casado E, Redondo A (2005). Breast carcinomas fulfill the Warburg hypothesis and provide metabolic markers of cancer prognosis. *Carcinogenesis*.

[27] Amon LM, Pitteri SJ, Li CI (2012). Concordant release of glycolysis proteins into the plasma preceding a diagnosis of ER^+^ breast cancer. *Cancer Research*.

[28] de Oliveira MF, Amoêdo ND, Rumjanek FD (2012). Energy and redox homeostasis in tumor cells. *International Journal of Cell Biology*.

[29] Ward PS, Thompson CB (2012). Metabolic reprogramming: a cancer hallmark even Warburg did not anticipate. *Cancer Cell*.

[30] Mentis AFA, Kararizou E (2010). Metabolism and cancer: an up-to-date review of a mutual connection. *Asian Pacific Journal of Cancer Prevention*.

[31] Zha X, Sun Q, Zhang H (2011). mTOR upregulation of glycolytic enzymes promotes tumor development. *Cell Cycle*.

[32] Menendez JA, Vellon L, Oliveras-Ferraros C, Cufí S, Vazquez-Martin A (2011). mTOR-regulated senescence and autophagy during reprogramming of somatic cells to pluripotency: a roadmap from energy metabolism to stem cell renewal and aging. *Cell Cycle*.

[33] Williams AC, Collard TJ, Paraskeva C (1999). An acidic environment leads to p53 dependent induction of apoptosis in human adenoma and carcinoma cell lines: implications for clonal selection during colorectal carcinogenesis. *Oncogene*.

[34] Pavlides S, Whitaker-Menezes D, Castello-Cros R (2009). The reverse Warburg effect: aerobic glycolysis in cancer associated fibroblasts and the tumor stroma. *Cell Cycle*.

[35] Guppy M, Leedman P, Zu X, Russell V (2002). Contribution by different fuels and metabolic pathways to the total ATP turnover of proliferating MCF-7 breast cancer cells. *Biochemical Journal*.

[36] Gatenby RA, Gawlinski ET, Gmitro AF, Kaylor B, Gillies RJ (2006). Acid-mediated tumor invasion: a multidisciplinary study. *Cancer Research*.

[37] Homem de Bittencourt PI, Peres CM, Yano MM, Hirata MH, Curi R (1993). Pyruvate is a lipid precursor for rat lymphocytes in culture: evidence for a lipid exporting capacity. *Biochemistry and Molecular Biology International*.

[38] Kondoh H, Lleonart ME, Bernard D, Gil J (2007). Protection from oxidative stress by enhanced glycolysis; a possible mechanism of cellular immortalization. *Histology and Histopathology*.

[39] Gatenby RA, Gillies RJ (2004). Why do cancers have high aerobic glycolysis?. *Nature Reviews Cancer*.

[40] Gillies RJ, Gatenby RA (2007). Adaptive landscapes and emergent phenotypes: why do cancers have high glycolysis?. *Journal of Bioenergetics and Biomembranes*.

[41] Biamonti G, Caceres JF (2009). Cellular stress and RNA splicing. *Trends in Biochemical Sciences*.

[42] Greijer AE, van der Groep P, Kemming D (2005). Up-regualtion of gene expression by hypoxia is mediated predominantly by hypoxia-inducible factor I (HIF-I). *Journal of Pathology*.

[43] Serkova N, Boros LG (2005). Detection of resistance to imatinib by metabolic profiling: clinical and drug development implications. *American Journal of PharmacoGenomics*.

[44] Hsu P, Sabatini D (2008). Cancer cell metabolism: Warburg and beyond. *Cell*.

[45] Dhahbi J, Kim H, Mote P, Beaver R, Spindler S (2004). Temporal linkage between the phenotypic and genomic responses to caloric restriction. *Proceedings of the National Academy of Sciences of the United States of America*.

[46] Lunt SY, Vander Heiden MG (2011). Aerobic glycolysis: meeting the metabolic requirements of cell proliferation. *Annual Review of Cell and Developmental Biology*.

[47] John S, Weiss JN, Ribalet B (2011). Subcellular localization of hexokinases I and II directs the metabolic fate of glucose. *PLoS ONE*.

[48] Mathupala SP, Ko YH, Pedersen PL (2009). Hexokinase-2 bound to mitochondria: cancer’s stygian link to the “Warburg effect” and a pivotal target for effective therapy. *Seminars in Cancer Biology*.

[49] Mathupala SP, Ko YH, Pedersen PL (2006). Hexokinase II: cancer’s double-edged sword acting as both facilitator and gatekeeper of malignancy when bound to mitochondria. *Oncogene*.

[50] Mendoza EE, Pocceschi MG, Kong X (2012). Control of glycolytic flux by AMP-activated protein kinase in tumor cells adapted to low pH. *Translational Oncology*.

[51] Ferguson EC, Rathmell JC (2008). New roles for pyruvate kinase M2: working out the Warburg effect. *Trends in Biochemical Sciences*.

[52] David CJ, Chen M, Assanah M, Canoll P, Manley JL (2010). HnRNP proteins controlled by c-Myc deregulate pyruvate kinase mRNA splicing in cancer. *Nature*.

[53] Shashni B, Sakharkar KR, Nagasaki Y, Sakharkar MK Glycolytic enzymes PGK1 and PKM2 as novel transcriptional targets of PPAR gamma in breast cancer pathophysiology.

[54] Venkatachalam G, Kumar AP, Sakharkar KR, Thangavel S, Clement MV, Sakharkar MK (2011). PPAR*γ* disease gene network and identification of therapeutic targets for prostate cancer. *Journal of Drug Targeting*.

[55] Lagana A, Vadnais J, Le PU (2000). Regulation of the formation of tumor cell pseudopodia by the Na(+)/H(+) exchanger NHE1. *Journal of Cell Science*.

[56] Putney LK, Denker SP, Barber DL (2002). The changing face of the Na^+^/H^+^ exchanger, NHE1: structure, regulation, and cellular actions. *Annual Review of Pharmacology and Toxicology*.

[57] Reshkin SJ, Bellizzi A, Caldeira S (2000). Na^+^/H^+^ exchanger-dependent intracellular alkalinization is an early event in malignant transformation and plays an essential role in the development of subsequent transformation-associated phenotypes. *The FASEB Journal*.

[58] Grinstein S, Dixon SJ (1989). Ion transport, membrane potential, and cytoplasmic pH in lymphocytes: changes during activation. *Physiological Reviews*.

[59] Bell SM, Schreiner SM, Schultheis PJ (1999). Targeted disruption of the murine NHE1 locus induces ataxia, growth retardation, and seizures. *American Journal of Physiology*.

[60] Pouyssegur J, Franchi A, Pages G (2001). pHi, aerobic glycolysis and vascular endothelial growth factor in tumour growth. *Novartis Foundation Symposium*.

[61] Turturro F, Friday E, Fowler R, Surie D, Welbourne T (2004). Troglitazone acts on cellular pH and DNA synthesis through a peroxisome proliferator-activated receptor *γ*-independent mechanism in breast cancer-derived cell lines. *Clinical Cancer Research*.

[62] Akram S, Teong HFC, Fliegel L, Pervaiz S, Clément MV (2006). Reactive oxygen species-mediated regulation of the Na^+^-H^+^ exchanger 1 gene expression connects intracellular redox status with cells’ sensitivity to death triggers. *Cell Death and Differentiation*.

[63] Kliewer SA, Lenhard JM, Willson TM, Patel I, Morris DC, Lehmann JM (1995). A prostaglandin J2 metabolite binds peroxisome proliferator-activated receptor *γ* and promotes adipocyte differentiation. *Cell*.

[64] Pelicano H, Carney D, Huang P (2004). ROS stress in cancer cells and therapeutic implications. *Drug Resistance Updates*.

[65] Gibellini L, Pinti M, Nasi M (2010). Interfering with ROS metabolism in cancer cells: the potential role of quercetin. *Cancers*.

[66] Storey KB (1996). Oxidative stress: animal adaptations in nature. *Brazilian Journal of Medical and Biological Research*.

[67] Janssen AML, Bosman CB, van Duijn W (2000). Superoxide dismutases in gastric and esophageal cancer and the prognostic impact in gastric cancer. *Clinical Cancer Research*.

[68] Kanbagli O, Ozdemirler G, Bulut T, Yamaner S, Aykaç-Toker G, Uysal M (2000). Mitochondrial lipid peroxides and antioxidant enzymes in colorectal adenocarcinoma tissues. *Japanese Journal of Cancer Research*.

[69] Punnonen K, Ahotupa M, Asaishi K, Hyöty M, Kudo R, Punnonen R (1994). Antioxidant enzyme activities and oxidative stress in human breast cancer. *Journal of Cancer Research and Clinical Oncology*.

[70] Cobbs CS, Levi DS, Aldape K, Israel MA (1996). Manganese superoxide dismutase expression in human central nervous system tumors. *Cancer Research*.

[71] Hileman EA, Achanta G, Huang P (2001). Superoxide dismutase: an emerging target for cancer therapeutics. *Expert Opinion on Therapeutic Targets*.

[72] Nishiura T, Suzuki K, Kawaguchi T (1992). Elevated serum manganese superoxide dismutase in acute leukemias. *Cancer Letters*.

[73] Senthil S, Veerappan RM, Ramakrishna Rao M, Pugalendi KV (2004). Oxidative stress and antioxidants in patients with cardiogenic shock complicating acute myocardial infarction. *Clinica Chimica Acta*.

[74] Weisberg SP, McCann D, Desai M, Rosenbaum M, Leibel RL, Ferrante AW (2003). Obesity is associated with macrophage accumulation in adipose tissue. *The Journal of Clinical Investigation*.

[75] Hajer GR, van Haeften TW, Visseren FL (2008). Adipose tissue dysfunction in obesity, diabetes, and vascular diseases. *European Heart Journal*.

[76] Kumar-Sinha C, Ignatoski KW, Lippman ME, Ethier SP, Chinnaiyan AM (2003). Transcriptome analysis of HER2 reveals a molecular connection to fatty acid synthesis. *Cancer Research*.

[77] Menendez JA, Mehmi I, Verma VA, Teng PK, Lupu R (2004). Pharmacological inhibition of fatty acid synthase (FAS): a novel therapeutic approach for breast cancer chemoprevention through its ability to suppress Her-2/neu (erbB-2) oncogene-induced malignant transformation. *Molecular Carcinogenesis*.

[78] Menendez JA, Oza BP, Atlas E, Verma VA, Mehmi I, Lupu R (2004). Inhibition of tumor-associated fatty acid synthase activity antagonizes estradiol- and tamoxifen-induced agonist transactivation of estrogen receptor (ER) in human endometrial adenocarcinoma cells. *Oncogene*.

[79] Hardy S, Langelier Y, Prentki M (2000). Oleate activates phosphatidylinositol 3-kinase and promotes proliferation and reduces apoptosis of MDA-MB-231 breast cancer cells, whereas palmitate has opposite effects. *Cancer Research*.

[80] Hardy S, El-Assaad W, Przybytkowski E, Joly E, Prentki M, Langelier Y (2003). Saturated fatty acid-induced apoptosis in MDA-MB-231 breast cancer cells. A role for cardiolipin. *The Journal of Biological Chemistry*.

[81] Desvergne B, Wahli W (1999). Peroxisome proliferators-activated receptors: nuclear control of metabolism. *Endocrine Reviews*.

[82] Medina-Gomez G, Gray S, Vidal-Puig A (2007). Adipogenesis and lipotoxicity: role of peroxisome proliferator-activated receptor *γ* (PPAR*γ*) and PPAR*γ*coactivator-1 (PGC1). *Public Health Nutrition*.

[83] Han L, Zhou R, Niu J, McNutt MA, Wang P, Tong T (2010). SIRT1 is regulated by a PPAR*γ*-SIRT1 negative feedback loop associated with senescence. *Nucleic Acids Research*.

[84] Giovannucci E, Harlan DM, Archer MC (2010). Diabetes and cancer: a consensus report. *CA Cancer Journal for Clinicians*.

[85] Liao S, Li J, Wang L, Zhang Y, Wang C (2010). Type 2 diabetes mellitus and characteristics of breast cancer in China. *The Asian Pacific Journal of Cancer Prevention*.

[86] Boyle P, Boniol M, Koechlin A (2012). Diabetes and breast cancer risk: a meta-analysis. *British Journal of Cancer*.

[87] Carpenter DO (2008). Environmental contaminants as risk factors for developing diabetes. *Reviews on Environmental Health*.

[88] Huang JT, Welch JS, Ricote M (1999). Interleukin-4-dependent production of PPAR-*γ* ligands in macrophages by 12/15-lipoxygenase. *Nature*.

[89] Nagy L, Tontonoz P, Alvarez JGA, Chen H, Evans RM (1998). Oxidized LDL regulates macrophage gene expression through ligand activation of PPAR*γ*. *Cell*.

[90] Berger J, Bailey P, Biswas C (1996). Thiazolidinediones produce a conformational change in peroxisomal proliferator-activated receptor-*γ*: binding and activation correlate with antidiabetic actions in db/db mice. *Endocrinology*.

[91] Lehmann JM, Moore LB, Smith-Oliver TA, Wilkison WO, Willson TM, Kliewer SA (1995). An antidiabetic thiazolidinedione is a high affinity ligand for peroxisome proliferator-activated receptor *γ* (PPAR*γ*). *The Journal of Biological Chemistry*.

[92] Lambe KG, Tugwood JD (1996). A human peroxisome-proliferator-activated receptor-*γ* is activated by inducers of adipogenesis, including thiazalidinedione drugs. *European Journal of Biochemistry*.

[93] Acton JJ, Black RM, Jones AB (2005). Benzoyl 2-methyl indoles as selective PPARgamma modulators. *Bioorganic & Medicinal Chemistry Letters*.

[94] Fair AM, Dai Q, Shu XO (2007). Energy balance, insulin resistance biomarkers, and breast cancer risk. *Cancer Detection and Prevention*.

[95] Pisani P (2008). Hyper-insulinaemia and cancer, meta-analyses of epidemiological studies. *Archives of Physiology and Biochemistry*.

[96] Belfiore A, Genua M, Malaguarnera R (2009). PPAR-*γ* agonists and their effects on IGF-I receptor signaling: implications for cancer. *PPAR Research*.

[97] Järvinen HY (2004). Thiazolidinediones. *The New England Journal of Medicine*.

[98] Burstein HJ, Demetri GD, Mueller E, Sarraf P, Spiegelman BM, Winer EP (2003). Use of the peroxisome proliferator-activated receptor (PPAR) *γ* ligand troglitazone as treatment for refractory breast cancer: a phase II study. *Breast Cancer Research and Treatment*.

[99] Kawa S, Nikaido T, Unno H, Usuda N, Nakayama K, Kiyosawa K (2002). Growth inhibition and differentiation of pancreatic cancer cell lines by PPAR*γ* ligand troglitazone. *Pancreas*.

[100] Mueller E, Smith M, Sarraf P (2000). Effects of ligand activation of peroxisome proliferator-activated receptor *γ* in human prostate cancer. *Proceedings of the National Academy of Sciences of the United States of America*.

[101] Shimada T, Kojima K, Yoshiura K, Hiraishi H, Terano A (2002). Characteristics of the peroxisome proliferator activated receptor *γ* (PPAR*γ*) ligand induced apoptosis in colon cancer cells. *Gut*.

[102] Ricote M, Li AC, Willson TM, Kelly CJ, Glass CK (1998). The peroxisome proliferator-activated receptor-*γ* is a negative regulator of macrophage activation. *Nature*.

[103] Kintscher U, Lyon CJ, Law RE (2004). Angiotensin II, PPAR-gamma and atherosclerosis. *Frontiers in Bioscience*.

[104] Helbig G, Christopherson KW, Bhat-Nakshatri P (2003). NF-*κ*B promotes breast cancer cell migration and metastasis by inducing the expression of the chemokine receptor CXCR4. *The Journal of Biological Chemistry*.

[105] Sung B, Park S, Yu BP, Chung HY (2006). Amelioration of age-related inflammation and oxidative stress by PPAR*γ* activator: suppression of NF-*κ*B by 2, 4-thiazolidinedione. *Experimental Gerontology*.

[106] Sung B, Park S, Yu BP, Chung HY (2004). Modulation of PPAR in aging, inflammation, and calorie restriction. *The Journals of Gerontology A*.

[107] Houseknecht KL, Cole BM, Steele PJ (2002). Peroxisome proliferator-activated receptor gamma (PPAR*γ*) and its ligands: a review. *Domestic Animal Endocrinology*.

[108] Ricote M, Li AC, Willson TM, Kelly J, Glass CK (1998). The peroxisome proliferators activated receptor-gamma is a negative regulator of macrophage activation. *Nature*.

[109] Kerppola TK, Luk D, Curran T (1993). Fos is a preferential target of glucocorticoid receptor inhibition of AP-1 activity in vitro. *Molecular and Cellular Biology*.

[110] Kamei Y, Xu L, Heinzel T (1996). A CBP integrator complex mediates transcriptional activation and AP-1 inhibition by nuclear receptors. *Cell*.

[111] Glass CK, Rosenfeld MG (2000). The coregulator exchange in transcriptional functions of nuclear receptors. *Genes & Development*.

[112] Tyagi S, Gupta P, Saini AS, Kaushal C, Sharma S (2011). The peroxisome proliferator-activated receptor: a family of nuclear receptors role in various diseases. *Journal of Advanced Pharmaceutical Technology & Research*.

